# External quality assessment study for ebolavirus PCR-diagnostic promotes international preparedness during the 2014 – 2016 Ebola outbreak in West Africa

**DOI:** 10.1371/journal.pntd.0005570

**Published:** 2017-05-01

**Authors:** Heinz Ellerbrok, Sonja Jacobsen, Pranav Patel, Toni Rieger, Markus Eickmann, Stephan Becker, Stephan Günther, Dhamari Naidoo, Livia Schrick, Kathrin Keeren, Angelina Targosz, Anette Teichmann, Pierre Formenty, Matthias Niedrig

**Affiliations:** 1 Centre for Biological Threats and Special Pathogens, Robert Koch Institute, Berlin, Germany; 2 Department of Virology, Bernhard-Nocht-Institute for Tropical Medicine, Hamburg, Germany; 3 Institute of Virology, Philipps University, Marburg, Germany; 4 Infectious Hazard Management department World Health Organization, Geneva, Switzerland; University of California, Los Angeles, UNITED STATES

## Abstract

During the recent Ebola outbreak in West Africa several international mobile laboratories were deployed to the mainly affected countries Guinea, Sierra Leone and Liberia to provide ebolavirus diagnostic capacity. Additionally, imported cases and small outbreaks in other countries required global preparedness for Ebola diagnostics. Detection of viral RNA by reverse transcription polymerase chain reaction has proven effective for diagnosis of ebolavirus disease and several assays are available. However, reliability of these assays is largely unknown and requires serious evaluation. Therefore, a proficiency test panel of 11 samples was generated and distributed on a global scale. Panels were analyzed by 83 expert laboratories and 106 data sets were returned. From these 78 results were rated optimal and 3 acceptable, 25 indicated need for improvement. While performance of the laboratories deployed to West Africa was superior to the overall performance there was no significant difference between the different assays applied.

## Author summary

For the highly infectious and deadly ebolavirus disease (EVD) to date neither specific treatment nor vaccines are available. Rapid and adequate isolation of patients is the only option to contain and to combat spreading of the disease. Reliable and sensitive diagnosis that allows efficient identification of infected individuals is a pre-requisite for outbreak management. External Quality Assurance (EQA) studies are a vital tool to assess individual diagnostic laboratory performance particularly important during the outbreak of novel emerging infections. Therefore, a panel of inactivated ebolavirus samples was generated in order to perform an EQA for ebolavirus diagnostic during the recent outbreak in West Africa to assess performance of mobile laboratories sent to the outbreak countries from different parts of the world. Further, the panel was provided to laboratories in other parts of the world to improve global preparedness in case EVD would spread through international travel or evacuation of infected international staff members deployed to West Africa to fight the disease. While 73.6% of all results reported during this study were rated optimal the performance of the laboratories from the outbreak countries was even better with 82.1% of the results rated optimal.

## Introduction

The Ebola outbreak in West Africa that started in December 2013 in the southeast of Guinea [1] has developed into the largest yet documented outbreak. While the human infection initiating this outbreak most likely was a zoonotic bat to human transmission of a Zaire ebolavirus variant [1, 2] subsequent spreading of ebolavirus disease (EVD) occurred via infected bodily fluids through close human-to-human contact. The core area of the outbreak was limited to the three most affected countries Guinea, Liberia, and Sierra Leone. The virus variant meanwhile has been named Makona (EBOV/Mak) after the Makona River in the Guinea/Liberia/Sierra Leone border region [3]. Close to 29 000 individuals were infected with EBOV/Mak and more than 11 300 died from EVD as of March 27, 2016. But imported cases and small outbreaks were also reported in Mali, Nigeria, Senegal, Europe and the United States [4]. EVD symptoms which can comprise high fever, nausea, vomiting, diarrhea, exanthema, coughing, and hemorrhage are highly unspecific, in particular in a region where malaria is highly endemic and where also other infectious diseases occur that might interfere with a clinical diagnosis of EVD [5]. Therefore, a quick and reliable diagnostic of suspected patients is of high priority to identify, isolate and treat infectious patients. Detection of viral RNA by reverse transcription polymerase chain reaction (RT-PCR) has proven effective for diagnosis of ebolavirus infection from acute cases since serology is only useful in the later stage of illness.

Several ebolavirus-specific RT-PCR assays have been published and commercial assays are available as well [6]. However the quality of these assays in particular regarding sensitivity and specificity are largely unknown and require a serious evaluation.

External Quality Assurance (EQA) studies are a vital tool to assess individual diagnostic laboratory performance and became especially important to assess technical capacities during outbreaks of novel emerging infections. Participants could benchmark the quality of their diagnostic performance, identify possible weaknesses and improve their diagnostic capabilities accordingly, allowing the most accurate EVD diagnostic in order to rapidly identify and isolate new cases.

## Material and methods

### Participants

Participating laboratories from Africa were nominated by the WHO Geneva office with a strong focus on the outbreak countries. Ebola-PCR EQA panels were also distributed among the members of the European Network for Diagnostic of Imported Viral Diseases (ENIVD), the German National Laboratory Network for Diagnostic of Biothreat Agents (NaLaDiBa) and the Global Health Security Action Group Laboratory Network (GHSAG-LN). Participation was free of charge. Laboratories were coded and after evaluation of the results participants received a table with all data sets but only their own laboratory was identified.

### Preparation of inactivated virus stocks

Viruses were grown on Vero E6 cells. Supernatant was inactivated by heat treatment (1h, 56°C), subsequent gamma irradiation on dry ice at 25–30 kgray (Synergy Health Radeberg GmbH, Radeberg, Germany), and tested for inactivation by cultivation in tissue culture. Cultures were passaged three times on Vero E6 cells. In supernatants no replication of virus was detected by specific real-time RT-PCR, thus confirming absence of infectivity. Inactivated virus stocks were stored at -80°C until further use.

### Generation of ebola PCR EQA panels

**Fig 1:**
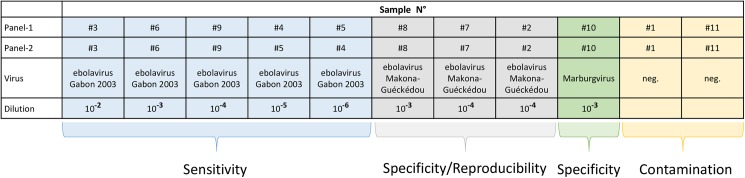
Panel composition for ebola EQA. The panel was composed of a serial dilution of Zaire ebolavirus isolate Gabon 2003 to test for sensitivity of diagnostic, a recent field isolate from West Africa (Makona-Guéckédou) to test for specificity and reproducibility, Marburgvirus to test for specificity and two negative samples with human plasma to test for contamination. Two panels (Panel-1 and Panel-2) were generated with identical dilution steps. Sample numbers, virus composition and corresponding dilution are shown.

To determine sensitivity of the ebolavirus diagnostic performed by the participating laboratories a 10-fold serial dilution of the Zaire ebolavirus (EBOV Gabon 2003, Genbank Acc. No. EF490230) preparation in distilled water and lyophylisation reagent (OPS Diagnostics, Lebanon, USA) was generated. Dilutions from 10^−2^ to 10^−6^ as well as 10^−3^ and 10^−4^ dilution steps of an early field isolate from the outbreak region Mak-C05 (GIN/2014/Makona-Guéckédou-C05, GenBank Acc. No. KJ660348), were included. To test for reproducibility the 10^−4^ sample of this isolate was included in duplicate. Marburgvirus isolate Popp (Genbank Acc.No. Z29337) was included as a 10^−3^ dilution of the virus stock. Two negative controls contained human plasma from blood donors. Aliquots of 100 μl of virus were freeze-dried together with 100 μl of 2x Lyophilization reagent (Ops diagnostics, NJ, USA) in 0.5ml glass vials with plugs (SP Industries, USA) in a freeze dryer (Epsilon 2-6D, Martin Christ Gefriertrocknungsanlagen GmbH, Germany). The samples of the panel were encoded with randomly distributed numbers from 1 to 11 (Fig 1) and stored at 4°C in the dark.

### Validation and shipment of panels

Sets of freeze-dried samples were pre-tested by three expert laboratories. Stability of samples was verified after 3 months at 4°C and an additional 4 weeks at room temperature (approx. 22°C) and reconfirmed after 6 months at 4°C. No obvious loss of genome copies was detected.

Due to the continuing demand for the Ebola EQA panel preparation of a second set of panels became necessary. Starting from the inactivated virus stocks used for panel-1 pre-dilutions identical to panel-1 were made. To allow joint evaluation of results great care was taken to prepare panel-2 according to the same specifications as panel-1. Therefore, both panels were pre-tested by real-time RT PCR side by side. While most corresponding samples of the 2 panels showed little variation of Cq values the final dilution of Zaire ebolavirus (10^−6^) for panel-2 was very close to the detection limit of the PCR assay. Therefore, from this dilution step only 1 out of 4 aliquots was positive (Cq 35) while 3 were negative (Cq 45). Similar results were obtained from the three pre-test sites. In addition, sample numbering between the final two dilution steps (10^−5^ and 10^−6^) were exchanged. Panel-2 was also stored at 4°C and stability was confirmed by PCR after one month and repeatedly up to 14 months.

Ebolavirus EQA panels were shipped with appropriate documentation in small zip-lock bags with desiccation bags at ambient temperature either with regular mail or by courier service.

## Results

Performance of molecular diagnosis of virus infection is based on specificity, sensitivity, and reproducibility of the applied assays and reliability largely depends on the prevention of cross-contamination. To test for these parameters a panel of samples was established from inactivated stocks of filoviruses (Fig 1). In order to speed up the preparation process a 10-fold serial dilution was established of a Zaire ebolavirus isolate (EBOV Gabon 2003) that previously had been inactivated and tested as an Ebola standard. This dilution series was used in the panel to test for sensitivity and to establish the limit of detection for the individual participant. At the time of conception of the EQA, only limited sequence information on the outbreak strain and potential sequence divergence from former ebolaviruses were available. Therefore, one first human isolate Mak-C05 from the current outbreak originating in Guéckédou, Guinea was included. Laboratories from Africa, and in particular field laboratories in the outbreak region were identified by WHO for participation in the EQA. The EQA panel was also offered to members of ENIVD, NaLaDiBA, and GHSAG-LN. In addition, the panel was distributed globally to interested laboratories responsible for ebolavirus diagnostic in their respective countries. Panels were always sent without any refrigeration, participants were asked to resolve the lyophilized material in 100 μl of sterile bidest. water prior to extraction and to handle the resolved samples as regular serum samples that potentially might contain ebolavirus. They were instructed to extract the entire sample and to analyze all samples for the presence of ebolavirus RNA genome according to their established protocol, to report their results directly to RKI, and to include information on the extraction method and PCR assay used.

**Fig 2:**
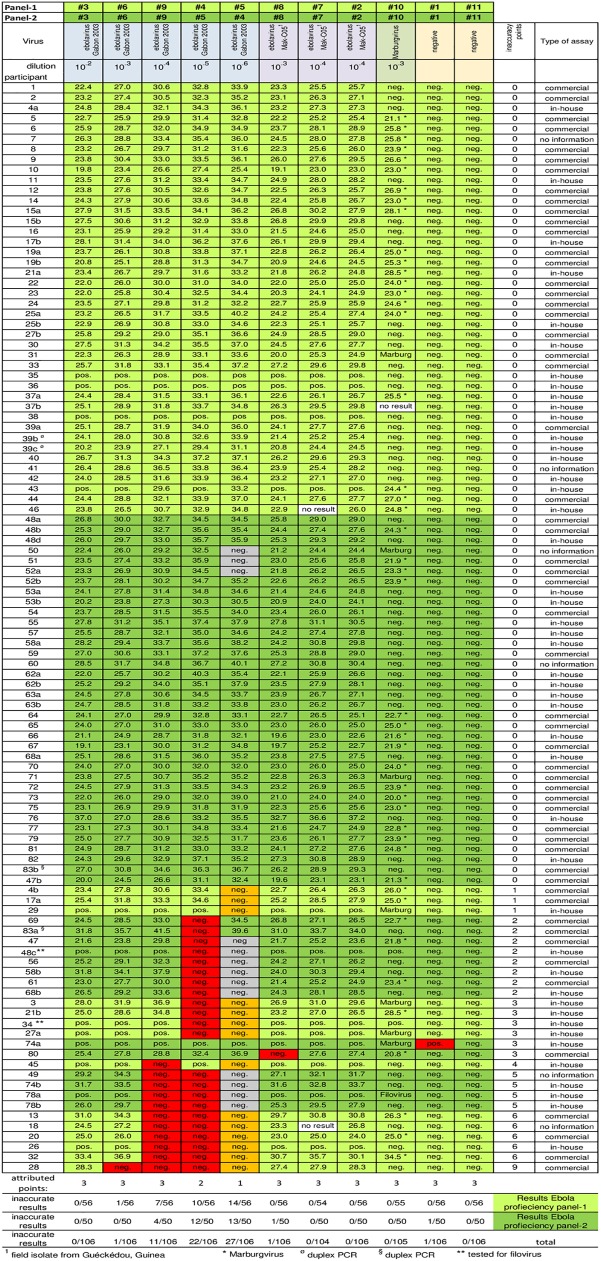
Representation of PCR results for ebola PCR-EQA. PCR results from all participants were collected, either as Cq values (when reported) or as positive or negative according to the report received. Sample type and dilution step of initial virus stocks are given at the top. The different categories approached in the EQA (sensitivity, specificity, reproducibility, contamination) are color coded according to Fig 1. Results received for panel-1 are shaded in light green. Results for panel-2 are shaded in dark green. False-positive and false-negative results are color coded. False-negative results for the lowest copy number (orange) were attributed 1 point (only panel-1), for the second lowest 2 points and all other false-negative as well as false-positive results received 3 points (all red). Coded participants are ranked according to their scores. The type of assay used for virus detection is shown in the column to the right. Three shipped panel sets had been incomplete and for the missing sample no result was obtained (white). For these samples as well as for the highest dilution for panel-2 (grey) no points were attributed.

A total of 106 data sets with PCR results for either Ebola PCR EQA panel-1 or panel-2 were returned by 83 labs in 42 countries (see acknowledgments). Of these 28 data sets were from 21 laboratories working in Sierra Leone and Guinea during the outbreak. While for 12 data sets samples were reported only as PCR-positive or PCR-negative all other 94 results were given with Ct values (Cq according to MIQE guidelines) [7] for samples tested positive. Participants were anonymized with numbers in the order of the arrival of results. Results for both panels were compiled in Fig 2. Several laboratories applied more than one assay to the panel and reported 2 or more results. These results were coded with the participant’s number discriminated by a, b, c and d, as required. While approx. half of the laboratories performed a diagnostic that also allowed identification of Marburgvirus the remaining participants reported this sample as “ebolavirus negative”. Since this study was specifically designed for the Ebola outbreak in West Africa participants were only asked to analyze the samples of the EQA panel for ebolavirus. Consequently a result for the Marburgvirus sample was also assessed “correct” if just reported as “ebolavirus negative”.

From the 106 data sets reported 55 (51.9%) were obtained with commercial assays (**Table 1**). From the remaining 51 data sets 26 results were obtained with 9 different in-house assays. Further 15 results were obtained with in-house assays but no reference was given. For 10 results no or too little information was available to determine if they had been obtained with commercial or with in-house assays.

**Table 1:** Ebola PCR assays used.

Assay	No. of results reported
**Commercial assays:**	
Altona diagnostic kits	47
Roche kit	2
Cepheid Xpert Ebola CE test*	2
Da' an detection kit	1
PuRuiKang Biotech LTD	1
Applied Biosystems (Invitrogen)	1
Federal Register (CDC)	1
**In-house assays:**	
Drosten et al. 2002 [8]	1
Fitzpatrick et al. 2015 [9]	2
Huang et al. 2012 [10]	2
Ogawa et al. 2011 [11]	2
Weidmann et al. 2004 [12]	2
Sanchez et al. 1999 [13]	3
Gibb et al. 2001 [14]	3
Trombley et al. 2010 [15]	5
Panning et al. 2007 [16]	6
no specific information	25
total	106

*Duplex assay

For the evaluation of this ebolavirus PCR EQA inaccurate results were rated with weighted inaccuracy points (Fig 2), 1 point for the highest dilution of the Zaire ebolavirus dilution series (marked orange), 2 points for the second highest dilution and for every other result not correctly analyzed 3 points (all marked red). The final dilution step (10^−6^) for Zaire ebolavirus in panel-2 (#4) was slightly more diluted than the corresponding sample in panel-1 (#5) and in the hands of the pre-test sites this sample was not reliably tested positive in RT qPCR. Therefore, a negative result for this sample was not rated as false-negative (marked in grey) and consequently was not attributed inaccuracy points. However, RT qPCR results for the second highest dilution (10^−5^) of panel-2 (#5) matched results for the second highest dilution from panel-1 (#4) in pre-tests. Therefore a false-negative result for this sample in panel-2 was also attributed 2 inaccuracy points. Results were ranked according to points. Identical scores were ranked according to arrival date.

78 data sets (73.6%) reported by 67 participants were rated optimal since all samples were identified correctly. Three additional results were rated acceptable since only the highest dilution of the panel had been analyzed false-negative (1 inaccuracy point) suggesting slightly reduced sensitivity for the assay performed. The remaining 25 results (23.6%) reported by 23 participants showed a clear need for improvement (2 to 9 inaccuracy points). However, out of these 23 participants 8 had reported additional results with an alternative assay that was rated optimal. This reduces the number of laboratories with urgent need for an improved assay to 15. Out of the 25 results not rated optimal or acceptable 10 were obtained with commercial, 13 with in-house assays. For the remaining 2 results there was no information available on the assay used.

All participants either identified the Marburgvirus sample correctly as PCR positive for Marburgvirus or reported it as “ebolavirus negative”. Only one participant reported a false-positive result for one of the two negative controls, all other participants identified the negative samples correctly.

**Fig 3:**
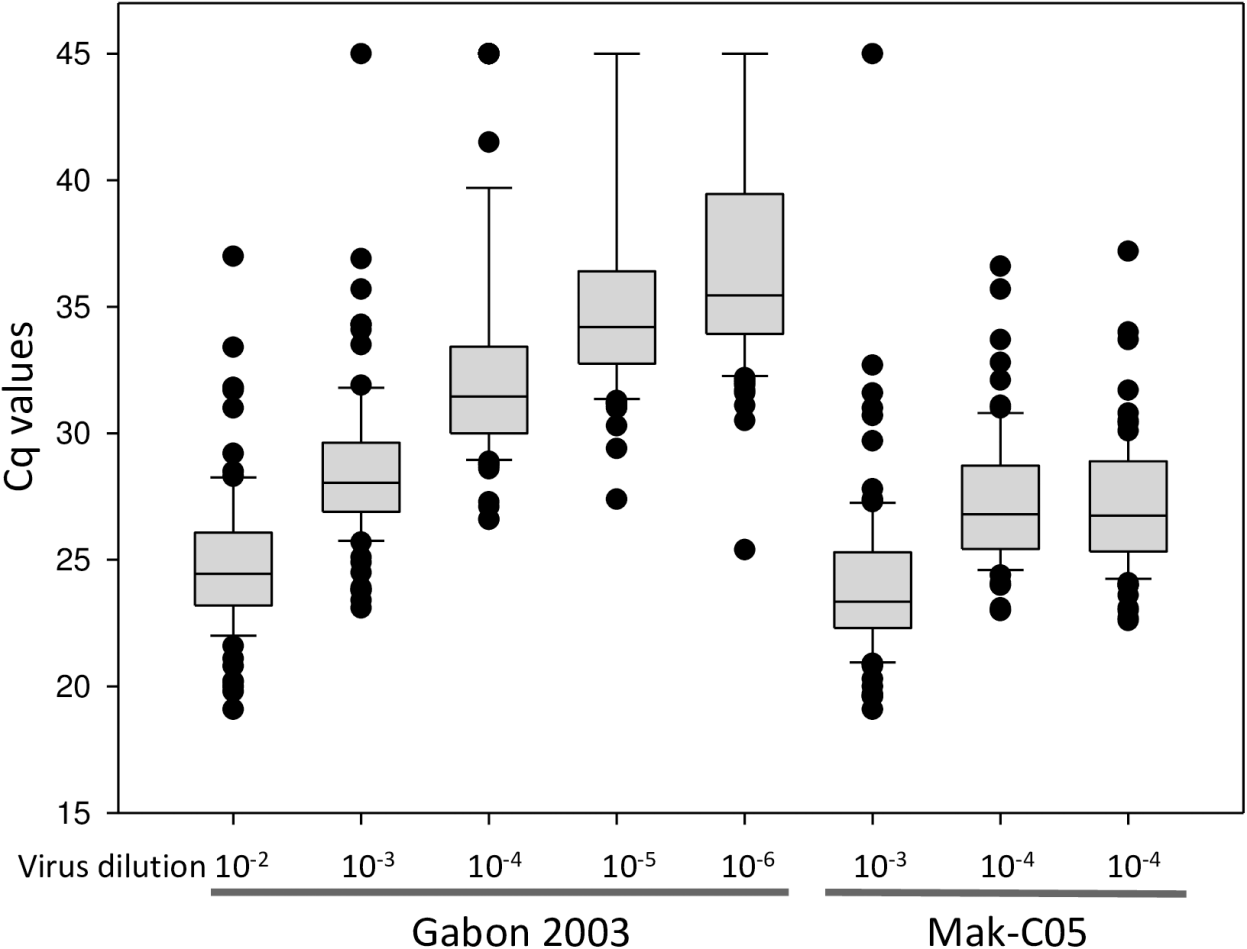
Box plot analysis of RT qPCR results. Box plot analysis displays the Cq values for RT qPCR from the 25 to 75 percentile for the dilution steps for Zaire ebolavirus and for the Makona field isolate. Cq values for each ebolavirus sample of the panel are represented by a box divided by the median line. Whiskers indicate the 5 to 95 percentile, individual outliers are indicated with dots.

Most participants used real-time RT-PCR assays for their analyses. While 102 data sets came with Cq values for positive samples only for 10 results copy numbers had been calculated and consequently quantification could not be considered for evaluation. Therefore, Cq values were taken as a semi-quantitative indicator and used for statistical analysis. For this purpose all negative results were translated into a Cq value of 45, since in many laboratories real-time PCR is routinely performed with a maximum of 45 cycles. For all positive samples Cq values were taken as reported but reduced to 1 position after decimal point if required. Since not all participants reported details on extraction procedure (e.g. extraction kit, elution volume) and volume of purified RNA applied to the PCR reaction this approach introduces an inaccuracy. Further, a few laboratories did not follow all the instructions provided with the panel and they only extracted part of the sample or pre-diluted the sample prior to testing. However, most participants (as far as indicated in the reports) used the entire sample for extraction and took 1/10 to 1/12 of the extracted RNA for PCR analysis. Additional variations might have been introduced since several different PCR assays and different real-time PCR instruments were used. These parameters might to some extend affect amplification efficiency and Cq value obtained for a given sample. Despite these ambiguities box-plot analysis showed a good over-all correlation for the panel between dilution and the mean for Cq values while overall variation of Cq values for a sample in the panel increased with the degree of dilution seen by an increase in the size of the boxes representing the 25 to 75 percentile (Fig 3). Reproducibility of results was tested with the identical samples #2 and #7. Good reproducibility was seen for 93 data sets. For these the difference between Cq values was not more than 1. For the remaining 13 results only for 4 data sets the difference was more than 2.

## Discussion

This study was initiated in late summer of 2014 in the light of the rapidly developing ebolavirus outbreak in West Africa with already high numbers of cases, disastrous predictions for the future development, and under the threat of global spreading through international air travel [17]. At present identification can be achieved best with sensitive, reliable, and rapid diagnostic PCR assays [18]. While WHO aimed at evaluating various laboratories that had been deployed to the outbreak countries in West Africa in the combined international effort to fight the disease, an additional motivation of RKI, ENIVD, NaLaDiBa, GHSAG-LN and WHO was to promote preparedness and evaluate quality of ebolavirus diagnostic beyond the outbreak region and on a global scale by supporting the development of diagnostic capacities and improving capabilities to allow rapid diagnosis of potentially emerging suspect cases.

In infected individuals ebolavirus can be detected early in the course of the disease and rapidly reaches high viral loads. In the absence of reliable rapid tests the method of choice for diagnosis of an acute infection is molecular detection of the viral genome via RT-PCR. In particular detection of pre-symptomatic patients would be beneficial for epidemic control [19]. Even at the end of the outbreak molecular detection of ebolavirus is still needed. It has been shown that survivors of the disease still can shed virus over long periods of time through seminal fluids, vaginal secretions, and breast milk, as well as several other body fluids [20, 21, 22] which have the potential of triggering new infections and flare-ups in countries that previously had already been declared Ebola-free [23].

This EQA was designed for laboratories prepared to inactivate and handle suspect samples under BSL3 conditions to perform PCR diagnostic on EVD suspect individuals [6]. This type of diagnostic does not require infectious virus and can be performed on inactivated clinical specimens. While preparation of purified viral genomic RNA, e.g. with the Qiagen viral RNA kit, has the advantage of efficiently eliminating infectivity [24, 25] distribution of purified RNA to participants also has several disadvantages: i) important pre-analytical steps are omitted and purification of nucleic acids prior to RT-PCR analysis cannot be evaluated, ii) stability of shipped RNA is critical [26] and shipment on dry ice would considerably increase costs for the study. In contrast analysis of carefully inactivated viruses also controls the pre-analytical steps prior to RT-PCR. Indeed, for some results indicating a need for improvement of diagnostic performance the information sent by the participating laboratories clearly pointed to problems at the level of sample preparation and not to the PCR analysis itself. While heat-treatment and gamma-irradiation reliably inactivate infectious viruses [27] the genomic RNA is still protected from degradation through RNases within viral particles although the Ebolavirus morphology of the viral particles has been altered through the inactivation process (M. Laue, personal communication). This combined inactivation was used as a safe inactivation method that has been used for different viruses including hemorrhagic fever viruses in other EQA studies [28]. Lyophylization of the inactivated material allows long-term storage and shipment at ambient temperature. Our most recent test of an Ebola-EQA panel set after 14 months of storage at 4°C confirmed unchanged quality of the material even for samples with low copy numbers. The samples from these sets could still be used to spike human blood and semen for validation studies [29].

This EQA study for Ebola PCR-diagnostic was developed during the largest hemorrhagic fever outbreak reported to date [4] and which—with the imminent threat of global spreading—was an enormous challenge for international public health. This had multiple consequences for the development of this particular EQA. The principle of former EQAs organized by ENIVD always had been to keep individual performance of participants confidential. Performance was only revealed to the individual participant after final evaluation of all results. This principle of confidentiality was kept for the majority of participants. However, the laboratories operating in the outbreak countries have been coordinated and supported as well as nominated for participation in the EQA by the Emerging and Dangerous Pathogens Laboratory Network at WHO headquarters in Geneva. In order to allow inclusion into the global evaluation of the public health situation performance of all those laboratories working in the outbreak countries was also revealed to WHO which shared results with the Ministries of Health for the respective outbreak countries. Participants were alerted to this fact by including this information into the documents provided with the EQA panel.

Since one of the main intentions of this study was to evaluate the laboratories deployed to the outbreak countries it is interesting to note that from 28 data sets received from 21 laboratories operating in Guinea or Sierra Leone during the outbreak 23 (82.1%) were optimal while 5 (17.9%) results were lacking sensitivity. Since the initial virus load for most of suspect EVD patients diagnosed in the outbreak countries was quite high [30], sensitivity of detection methods is most likely not the most important issue in an outbreak situation for the majority of samples. However, alternative sampling methods required for collection of blood samples from small children or because of religious or cultural reservations might affect sensitivity of PCR diagnostic [31] and therefore might require the most sensitive performance of the analytical method for certain patients also in an outbreak situation. Compared to the overall performance in this study with 73.6% of optimal results performance of laboratories from the outbreak countries was better. This may not surprise considering that the majority of mobile laboratories had technical expertise with EVD and / or BSL3 practice. Also, these laboratories had built a solid working routine during the outbreak. And although laboratory staff was exchanged on a regular base established and improved protocols were passed on from group to group and were even further refined if the necessity occurred (H. Ellerbrok, personal communication). Also, initial protocols and working routines were established from experts in the field and staff scientists volunteering to serve in these laboratories were also skilled and highly motivated bringing a solid working routine from their home institutions. Therefore, the results from this study revealing such an elevated performance for the deployed laboratories also show that international cooperation is a role model how to handle an emergency in future outbreaks. The panel was useful to provide confidence to the Ministries of Health in the outbreak countries that the international deployed laboratories were technically accurate. Further, the panel allowed increase of testing capacity beyond the limited number of laboratories with prior experience in working with EVD.
